# Commentary: raised c-troponin levels as a sign of myocardial injury after COVID-19 vaccination in healthy individuals are worrying

**DOI:** 10.1186/s43044-024-00441-1

**Published:** 2024-02-01

**Authors:** Rainer Johannes Klement, Harald Walach

**Affiliations:** 1 Department of Radiation Oncology, Leopoldina Hospital, Robert-Koch-Strasse 10, 97422 Schweinfurt, Germany; 2 Next Society Institute, Kazimieras Simonavicius University, Vilnius, Lithuania; 3 Change Health Science Institute, Basel, Switzerland

**Keywords:** Cardiovascular adverse events, mRNA vaccines, Myocarditis, SARS-CoV-2, Side effects

## Abstract

**Background:**

Recently, Buergin et al. (Eur J Heart Fail 25(10):1871–1881, 2023 doi:10.1002/ejhf.2978) thoroughly measured a frequency of 2.8% elevated high-sensitivity cardiac troponin T levels, a sign of myocardial damage, after mRNA-1273 (Moderna) booster vaccinations. In their discussion, they claim that before vaccinations were available, the incidence and extent of myocardial damage associated with COVID-19 infection would have been much higher. We here scrutinize this claim based on empirical data.

**Main body:**

Burgin et al. have only cited papers in support of their claim which considered hospitalized COVID-19 patients. After extracting COVID-19 infection data from Germany and Switzerland and the expected frequency of elevated troponin levels after COVID-19 infection in both hospitalized and non-hospitalized individuals, we find that the extent of myocardial damage after vaccinating a considerable proportion of the general population is expected to be much higher than after natural infections.

**Conclusions:**

The claim that the extent of myocardial injury after COVID-19 infection would be higher than after vaccination is not supported by empirical evidence and therefore wrong. We conclude that cross-national systematic observational studies should be conducted that allow a more precise estimation of the risk–benefit ratio of COVID-19 mRNA vaccinations.

## Background

Soon after starting the global COVID-19 vaccination campaign in 2021, reports of vaccine-associated myocardial damage began to accumulate [1–3]. A German autopsy study on 25 persons who had died unexpectedly and within 20 days after COVID-19 vaccination identified acute myocarditis as the most probable cause of unexpected death in four cases [4]. A study evaluating 18(^18^F)-fluorodeoxyglucose (FDG) uptake in the myocardium on PET/CT images from 303 non-vaccinated and 700 vaccinated asymptomatic patients found a significantly higher tracer uptake in the vaccinated group (*p* < 0.001) which was consistently observed up to 180 days after the second vaccine dose and may indicate subclinical myocardial inflammation [5]. Jeet Kaur and colleagues evaluated the WHO-pharmacovigilance database and found odds ratios for cardiovascular adverse events associated with COVID-19 vaccinations against the background of side-effects reported for various other medications of 2.1 for cardiac arrest, 2.7 for acute myocardial infarction, 7.3 for increased D-dimers, and 2.6 for increased troponin [6]. In a systematic review of various side-effects of Covid-19 vaccinations, Gøtzsche and Demasi reported 10 publications that deal with myocarditis or pericarditis as potential side-effects [7]. Nevertheless, due to a lack of prospective studies, the incidence and extent to which myocardial injury occurs after COVID-19 vaccination has remained largely unknown.

Recently, however, Buergin and colleagues published a study in the *European Journal of Heart Failure,* in which they report the results of a careful prospective monitoring of signs for myocarditis after mRNA-1273 (Moderna) booster vaccinations in a cohort of hospital employees in Basel, Switzerland [8]. Three days after receiving the vaccine, 40 out of 777 vaccinated individuals exhibited high-sensitivity cardiac troponin T (hs-TnT) levels exceeding the upper laboratory norm (≥ 9 ng/L). Of those, 22 (2.8% of all vaccinated individuals) were judged as having developed mRNA-1273 vaccine-associated myocardial injury and displayed median hs-TnT levels of 13.5 ng/L (IQR: 9–18.8 ng/L). In the Discussion of their paper, Buergin et al. stated: “*Before the COVID-19 vaccine were available, the incidence and extent of myocardial injury associated with COVID-19 infection was much higher than observed in this active surveillance study after booster vaccination*” [8]. Here, we argue that this statement is a logical mistake and therefore wrong. It is wrong for two reasons: First, Buergin et al. compared the incidence of elevated hs-TnT after booster vaccination to the incidence of myocardial injury associated with COVID-19 infection in *hospitalized* patients only, without considering the incidence of myocardial injury in the much larger number of the *infected, but not hospitalized* population. Second, the extent of myocardial injury in the vaccinated population not only depends on the incidence, but also on the total number of subjects who received a vaccine; this number is much larger than the number of individuals who contracted a SARS-CoV-2 infection. We will also discuss the public health implications of the findings of Buergin et al. regarding the impact of the vaccination campaigns on myocarditis risk, taking the general population in Germany and Switzerland as an example.

## Main text

### General approach

Our general approach consists in showing that neither the incidence nor the extent of myocardial injury due to natural SARS-CoV-2 infections in the general population was higher than those expected as a consequence of the COVID-19 vaccination campaign.

Buergin et al. [8] cite a total of three papers in support for their statement cited above, yet all three papers consider only *hospitalized* patients [9–11]. These papers clarify that high levels of cardiac troponin are very likely the concomitant marker of preexisting problems. However, in order to compare the incidence and extent of myocardial injury after vaccination and after a natural COVID-19 infection properly, one has to take into account both hospitalized and non-hospitalized infected individuals. To this aim, we tried to estimate the incidence of elevated cardiac TnT concentrations after SARS-CoV-2 infection in hospitalized and non-hospitalized individuals combined and to compare it to the incidence reported by Buergin et al., which is 40/777 individuals with elevated hs-TnT levels (5.1%) or 22/777 individuals with both elevated hs-TnT levels and suspected myocardial injury [8]. In a second step, we used the findings from Buergin et al. to estimate the expected number of affected individuals developing elevated cardiac troponin levels after COVID-19 vaccination in the general population of Germany and Switzerland and compared those numbers to the expected number of individuals developing elevated troponin levels after natural infection.

### Data

We extracted the number of officially confirmed SARS-CoV-2 infections in 2020 as well as the number of persons hospitalized with COVID-19 from official data reported by the Robert-Koch-Institute in Germany (https://www.rki.de/DE/Content/InfAZ/N/Neuartiges_Coronavirus/Daten/Klinische_Aspekte.html, accessed August 17, 2023) and by the Swiss Federal Office of Statistics (https://dam-api.bfs.admin.ch/hub/api/dam/assets/23771995/master, accessed August 17, 2023). In order to obtain estimates for the percentage of individuals experiencing elevated TnT levels after SARS-CoV-2 infection, we searched Google Scholar (https://scholar.google.de/, accessed August 17, 2023) for relevant studies measuring troponin levels in both hospitalized and non-hospitalized patients using the search term “troponin COVID-19 SARS-CoV-2 infection non-hospitalized”. This resulted in only one prospective study conducted by Niedziela et al. [12], who reported elevated cardiac TnT levels (> 14 ng/ml) in 7% (6/86) of hospitalized and in 1.8% (2/114) of non-hospitalized Covid-19 patients, giving a total incidence of 8/200 = 0.04 (standard error = 0.035) for elevated TnT levels after SARS-CoV-2 infection. Of those patients, only two displayed signs of past myocardial injury [12]. Table 1 compares the results of this study to those of Buergin et al. The study by Niedziela et al. [12] already shows that hospitalized infected individuals are more likely to exhibit signs of myocardial damage than non-hospitalized infected individuals. Another paper containing an overview over published data which was cited by Buergin et al. estimated the incidence of elevated troponin in hospitalized patients at 20% [10].

**Table 1:** Number of individuals with elevated troponin T levels after vaccination or natural infection with SARS-CoV-2, respectively, according to the data of Buergin et al. [8] and Niedziela et al. [12]

Troponin *T* levels	Vaccinated	Naturally infected
Elevated	40	8
Not elevated	737	192

For estimating the extent of myocardial injury after COVID-19 vaccination, we extracted the number of vaccinated persons derived from the national Dashboard in the case of Switzerland (https://www.covid19.admin.ch/de/vaccination/doses; accessed August 17, 2023) and from a representative survey in Germany [13].

### Statistical analysis

To test for an association between elevated TnT levels (yes versus no) and the route that spike protein enters the body (vaccination versus natural infection), we used the data given in Table 1. We applied both Fisher’s exact test and the binomial proportion test [14]. To estimate and compare the extent of myocardial injury after COVID-19 vaccination in the general population with the extent of myocardial injury after SARS-CoV-2 infection, we performed simple quantitative estimations as shown in Table 2. All calculations were performed using *R* version 4.3.1, and the threshold for statistical significance was set to 0.01.

**Table 2:** Estimation of persons in the general population at risk for elevated troponin levels (as a sign for myocardial damage) after COVID-19 vaccination compared to the risk of elevated troponin levels caused by SARS-CoV-2 infection

Country	Population*	Vaccinated against Covid 19	SARS-CoV2 cases in 2020**	Hospitalized according to National data in 2020**	Elevated troponin at > 14 ng/l in 1.8% of non-hospitalized cases	Elevated troponin at > 14 ng/l in 7% of the hospitalized Covid-19 patients	Elevated troponin, estimated at 20% of hospitalized	Elevated troponin > 13.5 ng/l (after vaccination) in 2.8% of the vaccinated population
Germany	84.4 million	83%: 70.384 million	1,782,948	137,333	32,093	9613	27467	1.97 million
Switzerland	8.7 million	69.76%: 6.07 million	454,197***	40,893	8175	2862	8179	169,960

^*^According to official statistical data

^**^source: Robert-Koch-Institute, data from 23rd June 2023, https://www.rki.de/DE/Content/InfAZ/N/Neuartiges_Coronavirus/Daten/Klinische_Aspekte.html (accessed 17th August 2023) and Swiss Office of Statistics (https://dam-api.bfs.admin.ch/hub/api/dam/assets/23771995/master accessed 17th August 2023)

^***^Our World in Data https://ourworldindata.org/covid-cases?country=GBR~CAN~DEU~FRA accessed 18th August 2023

Elevated troponin levels after vaccination were estimated from the systematic observation of Buergin et al. and are given in the last column. The risk of elevated troponin levels in hospitalized and non-hospitalized persons with SARS-CoV-2 infection was derived from National data in Germany and Switzerland and the results reported by Niedziela et al. [5] and Mueller et al. [3], respectively

## Results and discussion

Both Fisher’s exact test (*p* = 0.586) and the binomial proportion test (*p* = 0.627) applied to Table 1 indicated that the null hypothesis of equal proportions of individuals with elevated TnT levels after vaccination and natural infection could not be rejected. In a sensitivity analysis, we only considered those 22 (out of 777) individuals for whom Buergin et al. suggested an association with post-vaccine myocardial injury, and those two (out of 200) individuals for whom Niedziela et al. [12] found signs of past myocardial damage besides elevated TnT levels. Fisher’s exact test still gave no reason to reject the null hypothesis (*p* = 0.198), but the binomial proportion test yielded an almost significant p-value of 0.0171, showing some evidence that the risk of myocardial injury after SARS-CoV-2 infection might be *lower* than after mRNA-1273 vaccination. Together, these results indicate that the incidence of elevated TnT levels after SARS-CoV-2 infection is not significantly higher than after mRNA-1273 vaccination, if infected individuals with only mild-moderate symptoms (not requiring hospitalization) are considered.

Regarding the second part of the statement of Buergin and colleagues, that the “*extent of myocardial injury associated with COVID-19 infection was much higher than observed in this active surveillance study after booster vaccination”*, Table 2 reveals that this is not true. Their error derives from the fact that they did not account for the comparatively small number of Covid-19 infections and the even smaller number of hospitalized patients compared to the large number of people who were submitted to the risks of vaccination side effects. One should also consider that the population in the Buergin study comprised healthy hospital workers and the adjudicative process that determined causality was rigorous and conservative. The figure derived from Niedziela and colleagues [12] refers to non-hospitalized Covid-19 patients seen in a hospital and, thus, is an overestimation of likely heart problems in SARS-CoV-2 cases. In fact, Moulson et al. had studied 3018 young, competitive athletes and found a low prevalence (0.5% to 3.0%) of definite, probable, or possible SARS-CoV-2 cardiac involvement [15]. These athletes had been tested positive for SARS-CoV-2 between September 1, 2020, and December 31, 2020, and subsequently underwent cardiac screening, including TnT testing after a median of 12 days. Of 2719 athletes in whom TnT levels were assessed, 24 (0.9%) displayed abnormal elevations, but none of them experienced an adverse cardiac event during follow-up [15].

These indications that SARS-CoV-2-associated cardiac complications are rare in young, healthy individuals in fact exacerbate our findings: While the risk of severe cardiomyopathy is, in the case of SARS-CoV-2 infection without vaccination, mainly found in the elderly and comorbid patients, the risk of myocarditis in the vaccinated is equally stratified across age groups, even in those that have a negligible risk for cardiomyopathy as a sequela of infection.

Our results are limited by the scarcity of studies prospectively measuring troponin levels and other signs of myocardial injury in both hospitalized and non-hospitalized individuals with SARS-CoV-2 infection. We resorted to the data from Niedziela et al. despite the fact that troponin levels were measured at a median time of 106 days after symptom onset, while in the study of Buergin et al. they were measured three days after vaccination. Furthermore, Buergin et al. employed hs-TnT testing, while Niedziela et al. [12] utilized conventional TnT assays. Nevertheless, the data from Moulson et al. [15] indicate that utilizing the data from Niedziela et al. as indications for myocardial injury are probably overestimates, which would only strengthen our conclusions. Finally, owing to lack of more data, we have assumed that the incidence of myocardial injury after mRNA-1273 booster vaccination derived by Buergin et al. would also apply to other vaccination numbers and manufacturers. However, even if the risk for myocardial injury may be somewhat lower after receiving a different COVID-19 vaccination (such as BNT162b2 from Pfizer-BioNTech [16]), our results remain qualitatively valid due to the large number of vaccinated individuals (Table 2).

## Conclusions

With their rigorous assessment of cardiac troponin concentrations as a marker for myocardial damage in a healthy population, Buergin et al. made an important contribution to the safety monitoring of COVID-19 vaccines based on the novel mRNA technology. However, as we showed with our calculations (Tables 1 and 2), Buergin et al. underestimated the public health implications of their own finding due to a logical mistake. We conclude that cross-national systematic observational studies should be conducted that allow a more precise estimation of the risk–benefit ratio. We observe that our original call for careful assessment of this risk–benefit ratio still holds [17].

## Data Availability

All data utilized in this article is published and can be retrieved from the citied sources.

## Abbreviations

COVID-19: Coronavirus disease 2019
SARS-CoV-2: Severe acute respiratory syndrome coronavirus type 2
